# A Dual Immunological Raman-Enabled Crosschecking Test (DIRECT) for Detection of Bacteria in Low Moisture Food

**DOI:** 10.3390/bios10120200

**Published:** 2020-12-04

**Authors:** Cheng Pan, Binbin Zhu, Chenxu Yu

**Affiliations:** Department of Agricultural and Biosystems Engineering, Iowa State University, Ames, IA 50011, USA; pcxpy@iastate.edu (C.P.); juliachu@iastate.edu (B.Z.)

**Keywords:** SERS, bacterial detection, low moisture foods

## Abstract

Among the physical, chemical and biological hazards that could arise with respect to food safety, bacterial contamination has been one of the main concerns in recent years. Bacterial contamination in low moisture foods (LMFs) was an emerging threat which used to draw less attention as LMFs were considered at low risk of such a hazard. Bacteria can survive in low moisture environments and cause foodborne diseases once they enter the digestive system. Common detection methods such as ELISA and PCR are not well suited to LMFs, as most of them operate under aqueous environments. In this study, a Dual Immunological Raman-Enabled Crosschecking Test (DIRECT) was developed for LMFs using a nano-scaled surface enhanced Raman scattering (SERS) biosensor platform and multivariate discriminant analysis with a portable Raman spectrometer. It could provide a limit of detection (LOD) of 10^2^ CFU/g of bacteria in model LMFs, with a detection time of 30–45 min. It has the potential to become a quick screening method for on-site bacteria detection for LMFs to identify food safety risks in real time.

## 1. Introduction

For many years, low moisture foods (LMFs) have been considered safe from microbial contamination because the low water content is supposed to prevent growth of microbes. LMFs are defined as foods that have less than 0.85 water activity, and most bacteria such as *Salmonella* and *E. coli* need water activity of at least 0.91 to grow [1]. However, bacteria can still survive in environments with low water activity (<0.85). Many cases of foodborne disease outbreaks have been linked to the contamination of LMFs by pathogenic bacteria, such as *Salmonella* spp. in peanut butter, spice and milk powder [2]; and Shiga toxin-producing *E. coli* (STEC) strains in nuts, flour, rice [2], etc. These bacteria can retain their viability for a long time in low moisture environments. Given the opportunity and suitable condition, they can start to grow inside the human body and cause different levels of illness. Studies have shown that long term survival of pathogenic bacteria like *Salmonella* spp. can be from days to years under low moisture environments, which may even increase their resistance to heat treatment [1]. Moreover, low moisture environments may improve the tolerance of these pathogenic bacteria to other stresses such as low pH, UV radiation, disinfectants [2]. The infectious dose for the pathogens in LMFs can be very low (10 to 100 CFU) [2], hence, even if only small number of viable bacterial cells exist in the LMF, they can still cause illness when consumed.

Prevention of biological contamination in foods are regulated by the FDA and USDA, and hazard analyses are critical for the preventive control of pathogen contamination in foods. For LMFs, if the Good Manufacturing Practices (GMP) are followed, most of the pathogens can be killed during heating and dehydration processes. However, chances still exist that some pathogens with high tolerance can survive after those treatments and pose as a food safety threat. Reported foodborne disease outbreaks in LMFs have been on the rise in the past decade; the main pathogen culprits are *Salmonella* spp., *Bacillus cereus*, *Cronobacter sakazakii* (formerly *Enterobacter sakazakii*), *Clostridium* spp., *E. coli O157:H7*, and *Staphylococcus aureus* [3].

Numerous methods have been developed to detect pathogens in foods including culture based detection [4,5], Enzyme Linked Immunosorbent Assay (ELISA) [5], Polymerase Chain Reaction (PCR) [5,6] and biosensor methods such as Surface Plasmon Resonance (SPR) [4,5,6,7], Nanoparticle enabled bacterial capturing and detection [8,9,10], Surface-Enhanced Raman Spectroscopy (SERS) [11], mechanical biosensors [12], and electrochemical sensors [13]. Most of existing methods based on molecular recognition or genetic sequencing operate in aqueous environments. To use them for LMFs, pathogens in the food samples typically need to be collected via washing steps, and the wash water is then subject to testing. In general, these methods are not suitable for detecting pathogens directly in LMFs.

**Figure 1:**
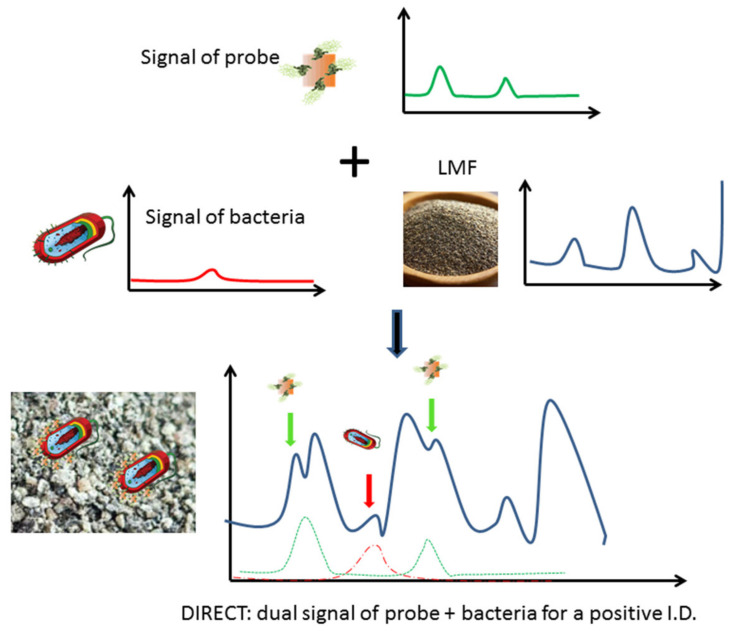
Scheme of the DIRECT assay, identification of peaks attributed to bacteria (red) and probes (green) yields a positive detection of contamination in LMF.

Among the variety of detection methods developed, surface enhanced Raman spectro-sensing (SERS) offers an appealing potential for LMF applications as it could provide a means to detect molecular recognition events in nonaqueous environments [14,15,16]. In SERS-based detection, a molecular recognition agent (MRA) such as antibody can be conjugated onto a nano-scale metallic structure (e.g., Au nanorods) to make a nanoprobe, and the binding of these nanoprobes to their molecular targets (e.g., epitopes on bacterial cell surface) can be detected and characterized via the Dual Immunological Raman-Enabled Crosschecking Test (DIRECT) developed in our lab [17,18], in which no washing/separation steps between target bound and unbound nanoprobes are needed. In the DIRECT scheme, gold nanoparticles are functionalized with Raman tags (e.g., 4-aminothiolphenol, or 4-ATP) and MRAs to make Raman molecular probes (RMPs) which would transduce probe signal. As the RMPs were applied to interrogate a bacterial-contaminated sample, they bound to target cell surface epitopes. These binding events could bring enough RMPs onto the cell surface, and Raman spectroscopic signals of the cell wall/extracellular matrix were therefore also enhanced due to their close vicinity to the nanoparticles (<10 nm), which were multiple orders of magnitude more intense than unenhanced signals (SERS enhancement is typically 10^5^–10^6^ for bacterial cells). The enhanced cell signal became detectable (unenhanced cell signal would be too weak to be detectable), and the superimposed dual signals (enhanced cell+probe signals) would yield a positive detection of the bacterial contamination, as shown in Figure 1. Without enough of the RMPs bound, signal from cells will not be enhanced enough to be detectable. Nonspecific binding of RMPs onto nontargets (other cells in the sample) will not create enough binding events needed for SERS enhancement, and hence will not create false positives. This DIRECT scheme has been shown to work effectively for the detection of waterborne bacteria at extremely low levels (1–10 CFU/mL) [17,18].

In this study, the DIRECT scheme was applied for the direct interrogation of LMF samples to achieve a rapid detection of bacteria in the LMFs at the 10^2−3^ CFU/g level of contamination within 30–45 min. Detection of bacteria in LMF is much more difficult than in water. As shown in Figure 1, different from water, which itself is a weak Raman scatterer and does not generate much background signal, LMFs themselves could be strong Raman scatterers and generate a very strong background Raman signal. When the contamination level is relatively low, such background could be overwhelmingly dominating. Hence, peaks associated with both cells and probes could be weak compared to the LMF peaks, and their identification would become a challenge which requires statistical analysis. In this work, two LMFs were selected as model food systems (black pepper powder and egg powder), as they represent the main food safety concerns at the consumer end (black pepper powder, in restaurants/dinners) and the production end (egg powder, as a protein additive in various processing lines). A non-pathogenic *E. coli* strain was chosen as a model bacterium to evaluate the limit of detection (LOD) of the method as it was easy to culture and manipulate to prepare spiked LMFs with different contamination levels. As the detection mechanism was based on molecular recognition of antibody–antigen interaction, it is straightforward to modify the platform for the detection of other pathogenic bacterial species.

## 2. Experiment Section

### 2.1. Reagents and Antibodies

Hexadecyltrimethylammoniumbromide (CTAB, 99%), ben-zyldimethylammoniumchloride hydrate (BDAC, 99%), sodium borohydride (99%), L-ascorbic acid, gold (III) chloride hydrate (>99%), silver nitrate (>99%), 4-aminothiophenol (4-ATP, >99%), sodium nitrite (>99%) and *E. coli* serotype polyclonal antibody were all purchased from Sigma-Aldrich. Black pepper powder was bought from Walmart. SERS-active slides were bought from Ocean Insight. Deionized water (18 MΩ) was used in all the experiments.

Polyclonal anti-*E. coli* antibodies (NB200-579) were purchased from Novus Biological (Centennial, CO, USA) for *E. coli* molecular detection. *E. coli* cultures (ATCC#25922) were provided by a colleague (Dr. Brehm-Stecher).

### 2.2. Fabrication and Functionalization of Gold Nanorods to Make SERS Nanoprobes

Gold nanorods (GNRs) with aspect ratio of 2–2.5 (Figure S1e) were synthesized via the seed-mediated growth method [19], Details of the procedure were reported elsewhere [17]. The GNRs were further functionalized by 4-aminothiophenol (4-ATP) [20,21], as illustrated in Figure S1. Briefly, 4 mL of 6 nM gold nanorods were reacted with 1 mL of 10 mM 4-ATP dissolved in acidic water (pH = 2) and the mixtures were stirred vigorously at 60 °C for 3 h. The solution was centrifuged and washed twice with 3 mM CTAB acidic aqueous solution to get rid of the unbound 4-ATP. Finally, 4-ATP functionalized GNRs were resuspended in 2.5 mL of acidic water (pH = 4).

*E. coli* antibody has an initial concentration of 4.5 mg/mL. A quantity of 0.5 mL *E. coli* antibody was divided into 10 aliquots (each was diluted with PBS buffer to 1 mL). For each conjugation run, 0.5 mL 4-ATP functionalized GNRs were mixed with 0.5 mL 1 mM NaNO_2_. Then, the mixture was incubated at 4 °C for 30 min; 200 µL from one antibody aliquot (45 μg antibodies) were added into the solution and incubated at 4 °C overnight. The mixture was then centrifuged (6000× *g*, 10 min, 4 °C) and washed twice to remove the unbound antibodies, then the pellet was resuspended in 0.5 mL 1× PBS buffer. The final concentration of the nanoprobe was ~5 nM. They remained stable for up to 1 month.

### 2.3. Bacterial Cell Culture and Sample Preparation

*E. coli* was incubated in Luria-Bertani medium broth at 37 °C for 18 h. The bacterial cells were then centrifuged and washed with PBS buffer twice and finally redispersed in PBS buffer. The final bacterial cell concentration was determined by optical density (OD) measurement at 600 nm (the concentration of the bacterial cells was ~10^9^ CFU/mL at OD = 1.0). The cell suspension was then diluted to 10^3^ CFU/mL. Quantities of 1 mL and 0.1 mL of the cell suspension were then mixed with 1 g of black pepper/egg powder samples to create bacterial spiked samples at 10^3^ CFU/g and 10^2^ CFU/g levels for test with the DIRECT assay alongside with the un-spiked samples. Each sample was mixed with 0.5 mL SERS nanoprobes and incubated at 4 °C for 30 min in an Eppendorf tube to allow probe–target binding.

### 2.4. One-Step Raman Spectroscopic Measurement

**Figure 2:**
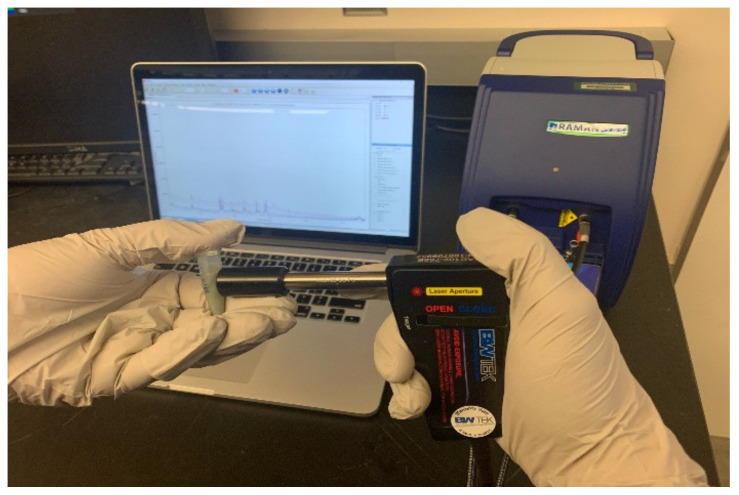
Illustration of the experimental setup.

As shown in Figure 2, the sample in the tube was then subjected to Raman measurement using a portable Raman spectrometer (i-Raman Plus, B&W Tek, Inc., Newark, DE, USA). The tube was put against the head of the fiber optical probe of the spectrometer, to allow the laser beam (15 mW, 785 nm NIR laser, 3 cm^−1^ spectral resolution, 400–2000 cm^–1^ range) to penetrate the plastic tube wall. Raman spectra of the sample were obtained directly in one single step. A 60 s integration time was used for spectral acquisition. A total of 15 spectra were collected from each sample to calculate an average spectrum, which was used for further analysis. All experiments were conducted in triplicates.

### 2.5. Spectral Data Processing

Using near infrared excitation (785 nm) effectively reduced the background sample auto-fluorescence. To further reduce the remaining fluorescence, a polynomial background subtraction method was implemented as described before [17,18]. The 10-point moving average method was used in this study to smooth the spectra to remove noise and other fine-scale variations. All spectra were then area-normalized for intensity consistency at the region between 500 cm^−1^ and 2000 cm^−1^. All data processing was conducted using R, a widely used language and software tool for statistical computing and graphics. Peak intensity was calculated based on the integration of the peak area.

## 3. Results and Discussion

### The DIRECT Assay for LMFs Contaminated with Model Bacteria

The mechanism of the self-referencing DIRECT assay was illustrated in Figure 1. Only specific binding of nanoprobes to targets (i.e., bacterial cells in LMF samples) would generate detectable dual SERS signals for a definitive positive readout for the bacteria. The DIRECT assay has been utilized to provide a 1–10 CFU/mL LOD for waterborne pathogens [18]. Similar to the earlier report, anisotropic gold nanorods with aspect ratios of ~2–2.5, as shown in Figure S1e, were also used in this study to generate specific SERS nanoprobes with conjugated *E. coli* antibodies via diazo chemistry through 4-Aminothiophenol (4-ATP) anchors. As shown in Figure S1a, after the conjugation, the nanoprobes showed stronger 4-ATP peaks as their fingerprints. The color of the nanoprobe aqueous solutions also changed after each step of the conjugation chemistry, indicating that the changes occurring on the nanoparticle surfacing were changing the optical properties of the nanoprobes, as shown in Figure S1b–d.

**Figure 3:**
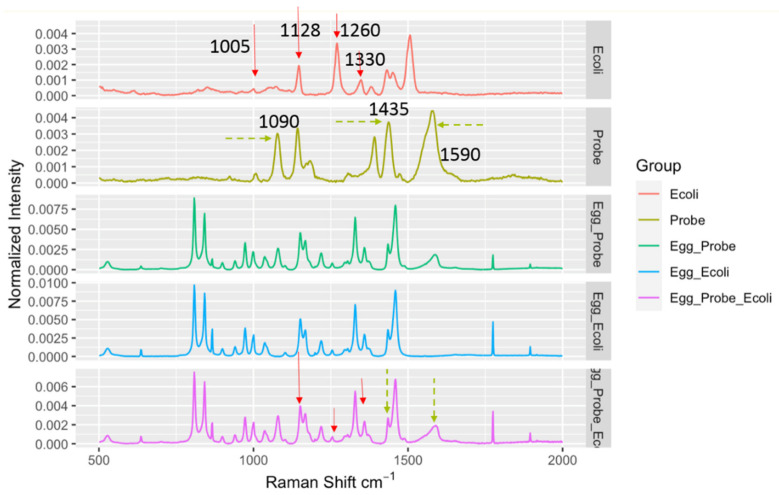
Spectral indicators showing presence of *E. coli* in spiked egg powder at 10^3^ CFU/g. Key Raman peaks were indicated by vertical lines, with red indicating *E. coli* relevance and green indicating probe relevance.

**Figure 4:**
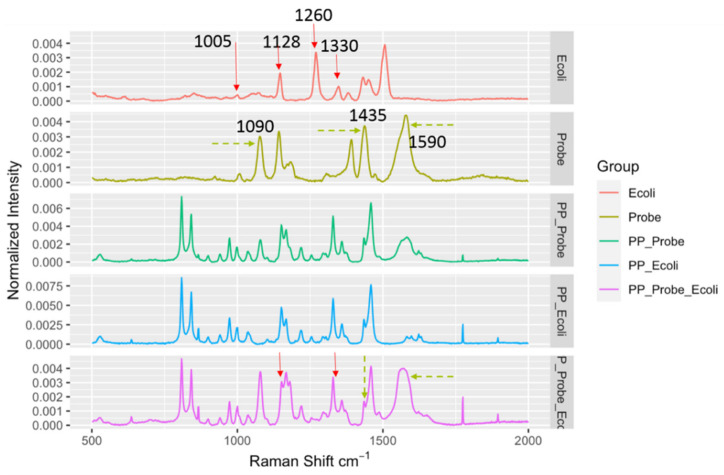
Spectral indicators showing presence of *E. coli* in spiked black pepper powder at 10^3^ CFU/g. Key Raman peaks were indicated by vertical lines, with red indicating *E. coli* relevance and green indicating probe relevance.

It should be noted that when the DIRECT assay was applied to LMFs, a quite unique challenge emerged compared to aqueous samples. In aqueous samples, the background was not a major interfering concern; the aqueous environment itself did not generate strong Raman signals to be superimposed on top of the signals generated by the target bacteria, regardless of whether they were bound to nanoprobes. For LMFs, the situation was completely different. The spectra taken from the LMF samples were always dominated by the peaks associated with the LMFs and the plastic Eppendorf tubes themselves. As shown in Figure 3, average spectra from egg_probe (un-spiked egg powder with probes added), egg_ecoli (spiked egg powder with no probe added) and egg_probe_ecoli (spiked egg powder with probe added) showed great similarity due to the common egg powder/tube contribution. Nonetheless, a close investigation of the spectra still suggested that in the egg_probe_Ecoli spectra, peaks could be identified that originated from the presence of nanoprobes binding to *E. coli* targets. The key Raman bands involved in the DIRECT scheme of target detection are listed in Table 1. The strong peaks at 1090 cm^−1^ and 1590 cm^−1^ were undoubtedly from the nanoprobes (4-ATP, as shown in Figure 3), which were weak on the spectra of egg_Ecoli when the probes were not present. The peaks at ~1128 cm^−1^ (carbohydrate) and ~1330 cm^–1^ (adenine-bearing molecules FAD, NAD, etc.) are typical of bacterial cell walls [22,23] which were enhanced by the SERS nanoprobes. It should be noted that the peak at 1005 cm^−1^, which could be assigned to phenylalanine, was very weak in the SERS spectrum of *E. coli* shown in Figure 3 and Figure 4, indicating that surface enhancement only affected the cell wall components [22]. These peaks could serve as an indication of the probe-cell binding in the DIRECT assay [17]. However, overlaps between these peaks and peaks from egg powder also were observed. It should be noted that some of the strong *E. coli* peaks (1260 cm^−1^ and 1509 cm^−1^) were not seen in the egg_Ecoli and egg_probe_ecoli samples at the 10^3^ CFU/g level of contamination. Apparently, the influence of the LMF itself (egg powder, a protein and lipid-rich food) was overwhelmingly strong. When nanoprobes bound to the *E. coli* cells in the spiked samples, only molecules on or right beneath the cell walls were sitting within the effective surface enhancement range, and peaks associated with other cell components would not be enhanced, and hence tended to become “invisible”, as evidenced by the disappearance of the phenylalanine peak at 1005 cm^−1^. In addition, the distribution of microbial cells in the LMFs would not be uniform, especially at low contamination levels, which also contributed to the weak signals from the cells. Only a couple of *E. coli* peaks could be seen in the DIRECT assay, which was consistent with our earlier work with waterborne pathogens. The presence of the dominant LMF background obviously complicated the identification of DIRECT signals by simple visual inspection of the spectral signals.

Similar results were also observed for another LMF, black pepper powder. The black pepper powder, as it was black, absorbed the laser energy much more effectively compared to the egg powder, and the energy absorption caused strong localized heating to occur, which, combined with the reduced scattered light, further complicated the SERS spectral measurements. Nonetheless, as shown in Figure 4, the DIRECT assay could provide spectral indicators for the detection of the presence of *E. coli* in the spiked samples. Similar to the results for egg powder, peaks at 1090 cm^−1^ and 1590 cm^−1^ could be attributed to the nanoprobes, and the peak at 1128 cm^−1^ appeared to be associated with the presence of *E. coli* in the spiked samples. It seems that the DIRECT scheme worked better for black pepper powder than for egg powder; further investigation is needed to reveal the reason(s) for this observation.

**Figure 5:**
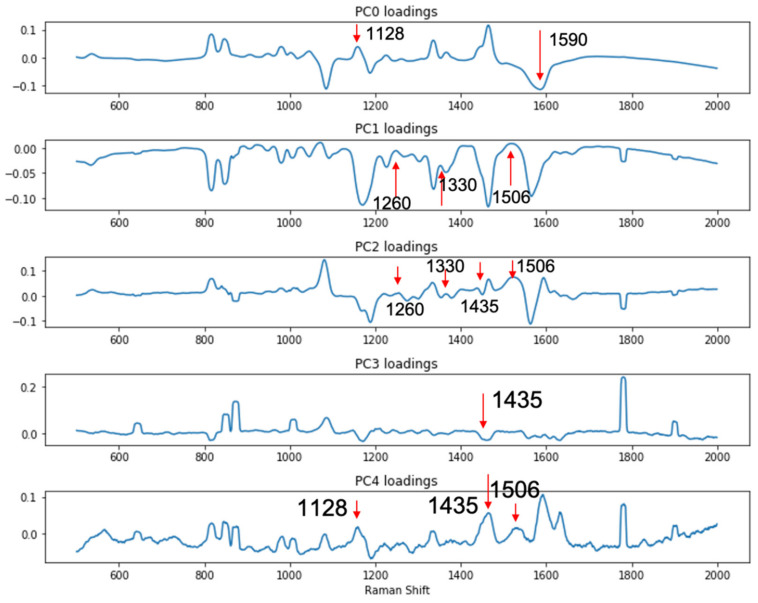
PC loadings showing peaks related to the detection of presence of *E. coli* in spiked black pepper powder at 10^3^ CFU/g.

Since the complication brought by the LMF backgrounds made simple visual inspection of the spectra unreliable and inconclusive, to better differentiate the signals from the DIRECT assay between contaminated (spiked) samples vs. control (unspiked) samples, a support vector machine (SVM)-based discriminant analysis was conducted to evaluate whether significant differences among the spectral signatures between the two groups of samples could be identified. The SVM model was constructed with principal components calculated from the spectral set of 80 randomly selected spectra (40 from each group, e.g., spiked black pepper powder vs. un-spiked black pepper powder); another set of 12 spectra from each group (also randomly selected) were used for testing the SVM model. Principal component (PC) analysis is routinely used for the reduction in complexity of spectral data to facilitate discriminant model construction. In this study, it was shown that the first five PCs accounted for over 90% of the total variance among the data sets for both egg powder and black pepper powder assays. As shown in Figure 5, the loadings of the first 5 PCs for the black pepper powder assays showed that these three PCs (1128 cm^−1^ and 1590 cm^−1^ peaks in PC0, 1260, 1330 and 1506 cm^−1^ peaks in PC1, 1260, 1330, 1435 and 1506 cm^−1^ peaks in PC2, 1435 cm^−1^ peaks in PC3, and 1128, 1435 and 1590 cm^−1^ peaks in PC4) were associated with the DIRECT identification of *E. coli* in the samples. Similar results were also obtained for egg powder samples, as shown in Figure S2.

Based on the study of the PC loadings, a discriminant model was constructed using the first five PCs. At the contamination level of 10^3^ CFU/g, for black pepper powder, the discriminant model provided a 97.2% differentiation accuracy between spiked and un-spiked black pepper powder for the testing set (12 spectra in the set). The black pepper powder was among the worst system for Raman spectroscopic analysis due to its strong light absorption; the high differentiation accuracy certainly demonstrated the potential of the DIRECT assay armed with PCA-SVM modeling.

The PCA-SVM model for the egg powder samples yielded reasonable differentiation results (at 87.5%), but not as good as for the black pepper powder, which was consistent with the less obvious results of the visual inspection of the spectra. The protein and lipid rich nature of the egg powder may contribute to these less-than-satisfactory results. At the 10^3^ CFU/g level, the contaminated black pepper powder samples could be clearly differentiated from un-spiked ones with the DIRECT assay with the help of PCA-SVM discriminant analysis. The PCA-SVM in this study was conducted with a limited dataset (40 spectra in the model set, 12 in the testing set); we deliberately chose this small dataset to see if the assay needs to be developed on the flight with limited data collection time, and how well it would work. It is fully understood that analysis with a limited dataset tends to be susceptible to overfitting, hence the 97.2% differentiation accuracy obtained could be biased. Further validation with a greater dataset is certainly needed. As reported earlier [18], multiplex epitope recognition should improve the accuracy of the DIRECT assay, which is to be explored in future work.

**Figure 6:**
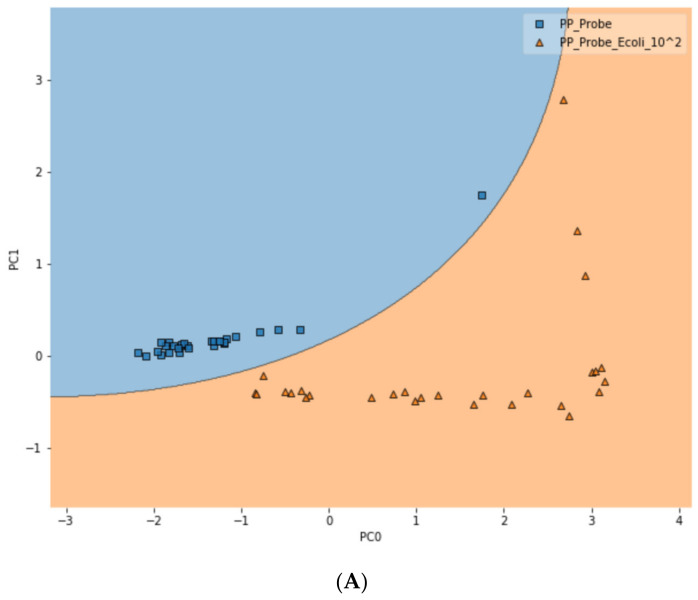

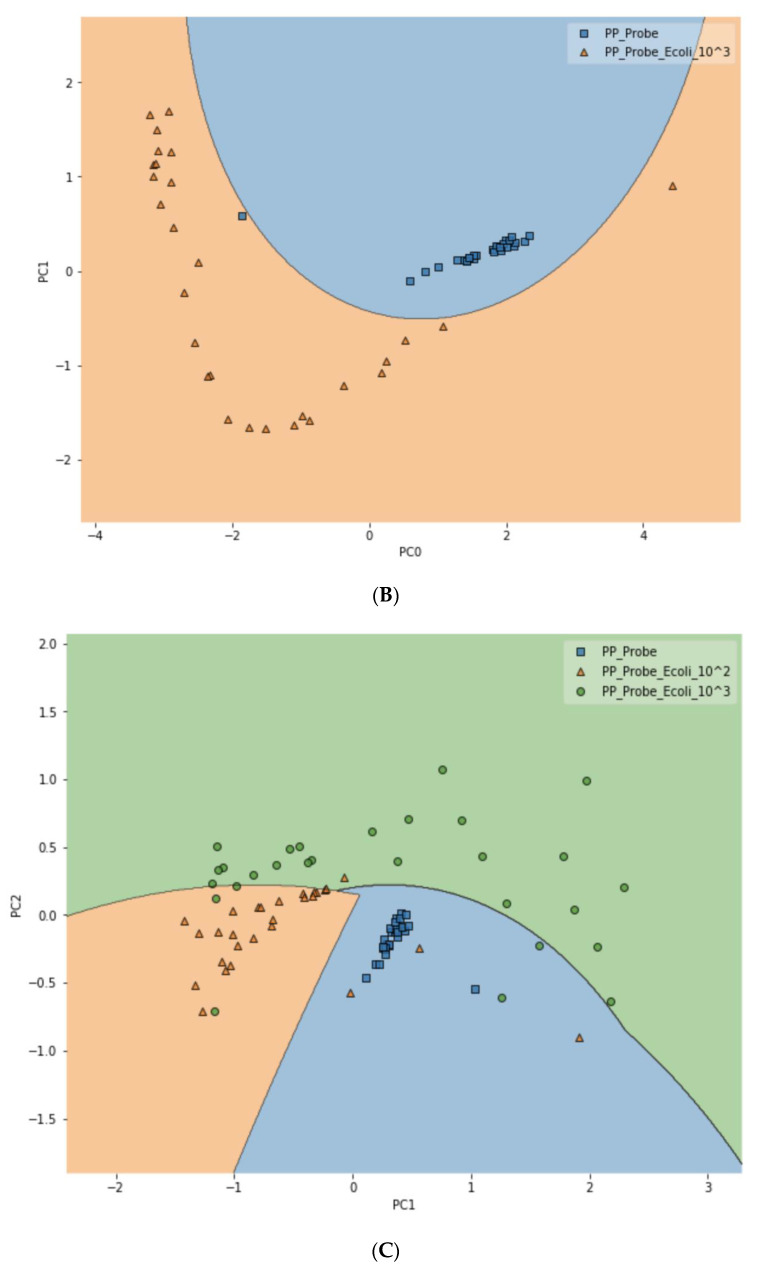
Classification of spiked vs. un-spiked black pepper powder using PCA-SVM discriminant modeling. (**A**) Contamination level of 10^2^ CFU/g; (**B**) contamination level of 10^3^ CFU/g; (**C**) differentiation among control, 10^2^ CFU/g and 10^3^ CFU/g samples.

To further test the limit of detection (LOD) of the DIRECT assay on LMFs, both LMF samples were also spiked with *E. coli* at the 10^2^ CFU/g level. For waterborne samples, it has been shown that the DIRECT assay can reach a LOD of 1–10 CFU/mL; but for the LMFs, that level of LOD would not be realistic. For 10^2^ CFU/g, however, the DIRECT assay appeared to work well, with only a slight decline in the differentiation accuracy. For black pepper powder, with the PCA-SVM model, the differentiation accuracy became 94.4% (compared to 97.2% at 10^3^ CFU/g); and for egg powder, it became 83.3% (compared to 87.5% at 10^3^ CFU/g). Figure 6 showed typical differentiation results by the PCA-SVM discriminant analysis that revealed clear separation of un-spiked vs. spiked black pepper powder samples at 10^2^ CFU/g contamination and 10^3^ CFU/g contamination. The SVM separation suggested that a semi-quantitative assay could potentially be developed that would allow a quick estimate of the contamination level. Similar results for egg powder samples are shown in Figure S3.

Ideally, the DIRECT assay result (i.e., the dual bands associated with both bacteria and nanoprobes) should be obvious with a simple visual inspection of the spectra, as has been shown for waterborne samples [17]. However, the presence of LMFs ruled out such a possibility as the original Raman signatures of the LMFs would always be much more visible than those of the bacteria and the probes, so the enhanced dual signals of the bacteria+probes could be hidden within the Raman spectrometer readings. As shown in Figure S4, for DIRECT spectral markers to become clear and a positive detection result to be made with certainty without discriminant analysis, the contamination level would need to reach 10^8^ CFU/g, which is no longer practically meaningful as contamination at such a level rarely occurs in reality. Another reason the enhancement was not strong at low contamination level is that the binding between the bacteria and the probes could be restricted due to low flowability of the materials in a low moisture environment. Using the multiplex epitope recognition scheme [18] could further improve the detection accuracy of target bacteria. Nonetheless, the results still suggested that the DIRECT assay can be used to qualitatively detect pathogens in LMFs with a very quick turnaround time to determine whether or not contamination has occurred, with a LOD of 10^2^ CFU/g, which is a big step forward for LMFs. However, further improvements are certainly needed to enhance the outcomes and to evaluate how well the scheme would work when multiple bacterial species are present simultaneously.

## 4. Conclusions

Using a DIRECT assay to detect bacteria in LMFs is feasible according to the experimental results of this work. For spices like black pepper, detection could be readily performed and good detection results could be obtained via statistical (i.e., SVM) discriminant analysis to differentiate contaminated samples at levels as low as 10^2^ CFU/g from bacteria-free control samples. Protein and lipid rich LMFs such as egg powder were more difficult to work with, and further improvement is needed via the multiplexing detection scheme to increase the sensitivity and specificity of the approach. Overall, the SERS-based DIRECT technique can deliver a rapid detection of bacteria in LMFs without damaging the format of the food compared to other conventional methods now available in the market, which in the future may offer an effective alternative for quick screening of LMFs for food safety concerns in industry.

## Figures and Tables

**Table 1:** Key band assignments of importance in the DIRECT scheme.

SERS Band	Assignment	Possible Origin for DIRECT Assay	Reference
1005 cm^−1^	Phenylalanine in protein	*E. coli* cells	[22]
1090 cm^−1^	C-S vibration, 4-ATP label	Probes	[18,20]
1128 cm^−1^	Carbohydrate	*E. coli* cells	[24,25]
1260 cm^−1^	Amide III, protein	*E. coli* cells	[22]
1330 cm^−1^	Adenine, FAD/NAD	*E. coli* cells	[22,23]
1435 cm^−1^	N=N stretching, diazonium	Probes, antibody-4-ATP linkage	[18,20]
1590 cm^−1^	C-C stretching, 4-ATP labels	Probes	[18,20]

## Supplementary Materials

The following are available online at https://www.mdpi.com/2079-6374/10/12/200/s1, Figure S1. Making of DIRECT molecular nanoprobes via functionalization of gold nanorods; Figure S2. PC loadings showing peaks related to the detection of presence of *E. coli* in spiked egg powder at 103 CFU/g; Figure S3. Classification of spiked vs. un-spiked black pepper powder using PCA-SVM discriminant modeling; Figure S4. Detection of *E. coli* in black pepper at different contamination levels with SERS probe.

## References

[1] Finn S., Condell O., McClure P., Amézquita A., Fanning S. (2013). Mechanisms of survival, responses and sources of Salmonella in low-moisture environments. Front. Microbiol..

[2] Rajagopal R. Best Practices for Pathogen Detection in Low Moisture Foods Food Quality and Safety, 26 January 2019. https://www.foodqualityandsafety.com/article/pathogen-detection-low-moisture-foods/3/?singlepage=1.

[3] Beuchat L.R., Komitopoulou E., Beckers H., Betts R.P., Bourdichon F., Fanning S., Joosten H.M., Ter Kuile B.H. (2013). Low–Water Activity Foods: Increased Concern as Vehicles of Foodborne Pathogens. J. Food Prot..

[4] Cho I.-H., Ku S. (2017). Current Technical Approaches for the Early Detection of Foodborne Pathogens: Challenges and Opportunities. Int. J. Mol. Sci..

[5] Priyanka B., Patil R.K., Dwarakanath S. (2016). A review on detection methods used for foodborne pathogens. Indian J. Med. Res..

[6] Wang Y., Salazar J. (2015). Culture-Independent Rapid Detection Methods for Bacterial Pathogens and Toxins in Food Matrices. Compr. Rev. Food Sci. Food Saf..

[7] Law J.W.-F., Ab Mutalib N.-S., Chan K.-G., Lee L.-H. (2015). Rapid methods for the detection of foodborne bacterial pathogens: Principles, applications, advantages and limitations. Front. Microbiol..

[8] Phillips R.L., Miranda O.R., You C.C., Rotello V.M., Bunz U.H. (2008). Rapid and efficient identification of bacteria using gold-nanoparticle-poly(para-phenyleneethynylene) constructs. Angew. Chem. Int. Ed. Engl..

[9] Xu X., Chen Y., Wei H., Xia B., Liu F., Li N. (2012). Counting bacteria using functionalized gold nanoparticles as the light-scattering reporter. Anal. Chem..

[10] Gu H., Ho P.-L., Tsang K.W.T., Wang L., Xu B. (2003). Using Biofunctional Magnetic Nanoparticles to Capture Vancomycin-Resistant Enterococci and Other Gram-Positive Bacteria at Ultralow Concentration. J. Am. Chem. Soc..

[11] Wang Y., Lee K., Irudayaraj J. (2010). Silver Nanosphere SERS Probes for Sensitive Identification of Pathogens. J. Phys. Chem. C.

[12] Arlett J., Myers E.B., Roukes M. (2011). Comparative Advantages of Mechanical Biosensors. Nat. Nanotechnol..

[13] Cinti S., Volpe G., Piermarini S., Delibato E., Palleschi G. (2017). Electrochemical Biosensors for Rapid Detection of Foodborne Salmonella: A Critical Overview. Sensors.

[14] Pahlow S., Meisel S., Cialla-May D., Weber K., Rösch P., Popp J. (2015). Isolation and identification of bacteria by means of Raman spectroscopy. Adv. Drug Deliv. Rev..

[15] Ravindranath S., Wang Y., Irudayaraj J. (2011). SERS driven cross-platform based multiplex pathogen detection. Sens. Actuators B Chem..

[16] Zhao X., Li M., Xu Z. (2018). Detection of Foodborne Pathogens by Surface Enhanced Raman Spectroscopy. Front. Microbiol..

[17] Xiao N., Wang C., Yu C. (2013). A Self-Referencing Detection of Microorganisms Using Surface Enhanced Raman Scattering Nanoprobes in a Test-in-a-Tube Platform. Biosensors.

[18] Wang C., Madiyar F., Yu C., Li J. (2017). Detection of extremely low concentration waterborne pathogen using a multiplexing self-referencing SERS microfluidic biosensor. J. Biol. Eng..

[19] Nikoobakht B., El-Sayed M. (2003). Preparation and Growth Mechanism of Gold Nanorods (NRs) Using Seed-Mediated Growth Method. Chem. Mater..

[20] Xiao N., Yu C. (2010). Rapid-Response and Highly Sensitive Noncross-Linking Colorimetric Nitrite Sensor Using 4-Aminothiophenol Modified Gold Nanorods. Anal. Chem..

[21] Alkilany A.M., Murphy C.J. (2010). Toxicity and cellular uptake of gold nanoparticles: What we have learned so far?. J. Nanoparticle Res. Interdiscip. Forum Nanoscale Sci. Technol..

[22] Efrima S., Zeiri L. (2009). Understanding SERS of bacteria. J. Raman Spectrosc..

[23] Zheng Y., Carey P.R., Palfey B.A. (2004). Raman spectrum of fully reduced flavin. J. Raman Spectrosc..

[24] Hamasha K., Mohaidat Q.I., Putnam R.A., Woodman R.C., Palchaudhuri S., Rehse S.J. (2013). Sensitive and specific discrimination of pathogenic and nonpathogenic *Escherichia coli* using Raman spectroscopy—A comparison of two multivariate analysis techniques. Biomed. Opt. Express.

[25] Liu Y., He L., Mustapha A., Li H., Hu Z.Q., Lin M. (2009). Antibacterial activities of zinc oxide nanoparticles against Escherichia coli O157:H7. J. Appl. Microbiol..

