# Supplementary material for: A Dual Immunological Raman-Enabled Crosschecking Test (DIRECT) for Detection of Bacteria in Low Moisture Food
Source: Biosensors (Basel). 2020 Dec 4;10(12):200. doi: 10.3390/bios10120200 (PMC7761983; doi:10.3390/bios10120200)
Supplement: Supplementary file 1 [file biosensors-10-00200-s001.pdf]

biosensors

MDPI

## Supplement materials

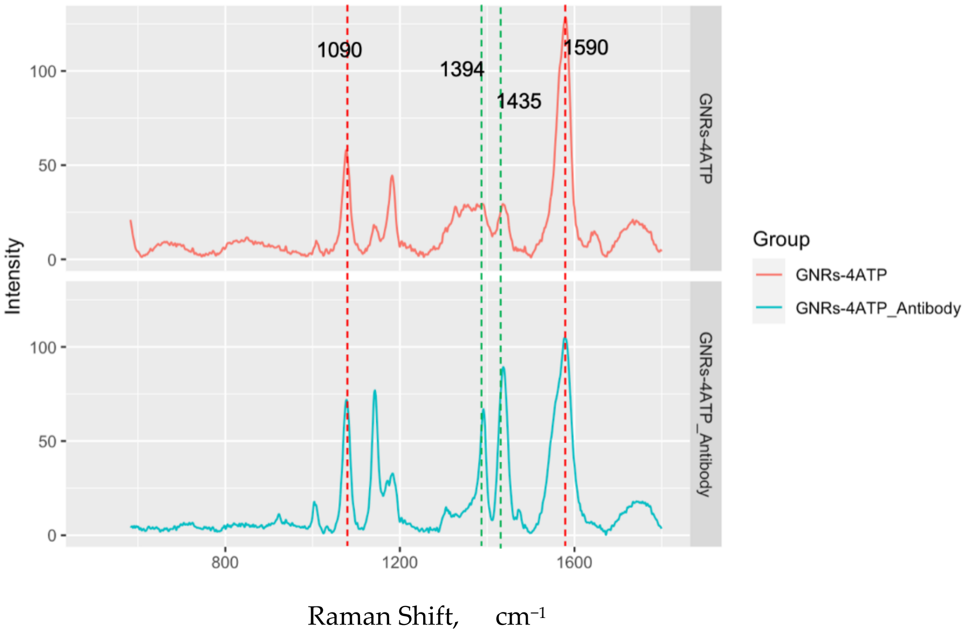


(a)

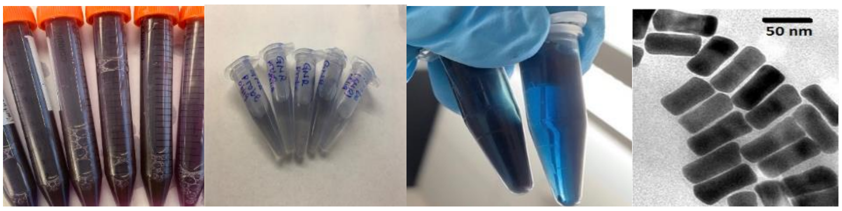


(b) (c) (d) (e)

**Figure S1.** Making of DIRECT molecular nanoprobes via functionalization of gold nanorods. (a). Raman spectra; (b). Gold Nanorods (aspect ratio≈2); (c). GNRs-4ATP; (d). GNRs Final Probes; (e). TEM image of the GNRs.

Fig. S1 shows the Raman spectra of 4-ATP-coated GNR and anti-E. coli antibody-conjugated GNR. A layer of 4-ATP molecules were anchored on the surface of the GNR due to Au-S bonding. As shown in fig S1a, band at 1090 $cm^{-1}$ is the stretching vibration of C-S bond and band at 1590 $cm^{-1}$ is the C-C stretching vibration of benzene ring in 4-ATP [1-5]. The appearance of these bands indicated the successful replacement of CTAB with 4-ATP on the GNR surface. Another notable difference between the spectra of pure 4-ATP and that of 4-ATP labeled Ag-cube is the intensity of 4-ATP characteristic peak at 1590 $cm^{-1}$. The apparent enhancement of the mode at 1590 $cm^{-1}$ can be ascribed to a charge transfer between the metal and the 4-ATP molecules [6], further confirms the binding of 4-ATP to the GNR surfaces.

The Ag-4-ATPs were then reacted with nitrite ions in acid condition to form diazonium salt, which subsequently reacted with histidine residues of the antibodies. The stengtherning of the 1394 $cm^{-1}$ and 1435 $cm^{-1}$ diazonium peaks (N=N stretching) proved the conjugation of the antibodies.

Panels b-d showed the change of color of the GNRs after each step of the surface chemical modification.

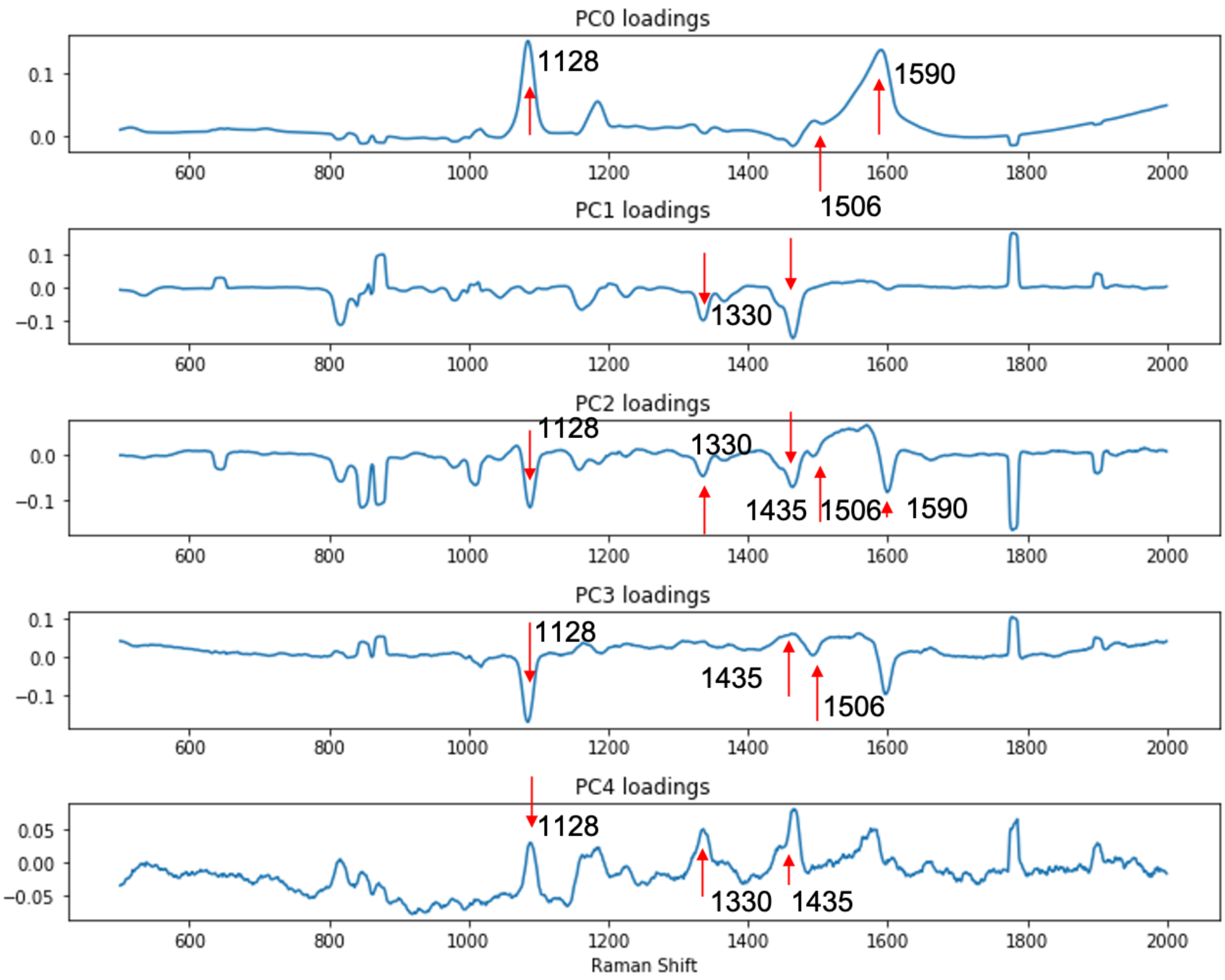


**Figure S2.** PC loadings showing peaks related to the detection of presence of E. coli in spiked egg powder at $10^3$ CFU/g.

Key peaks that originated from bacterial cells and probes could be identified in PC loadings, similar to what has been reported for spiked black pepper samples as discussed in the main text.

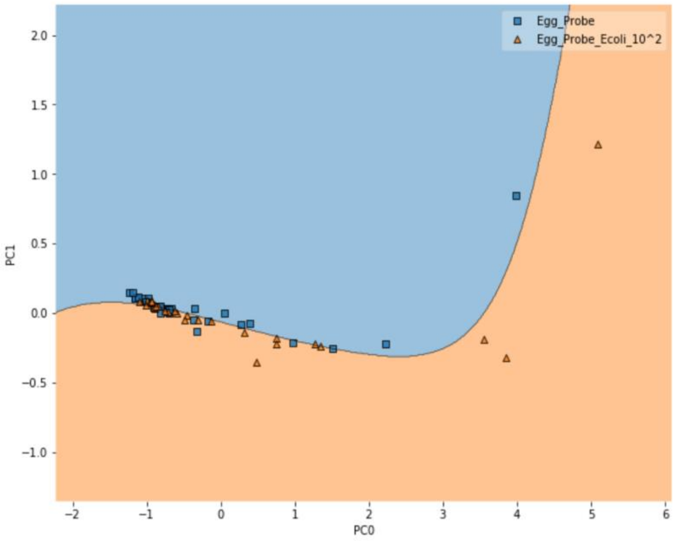


(A)

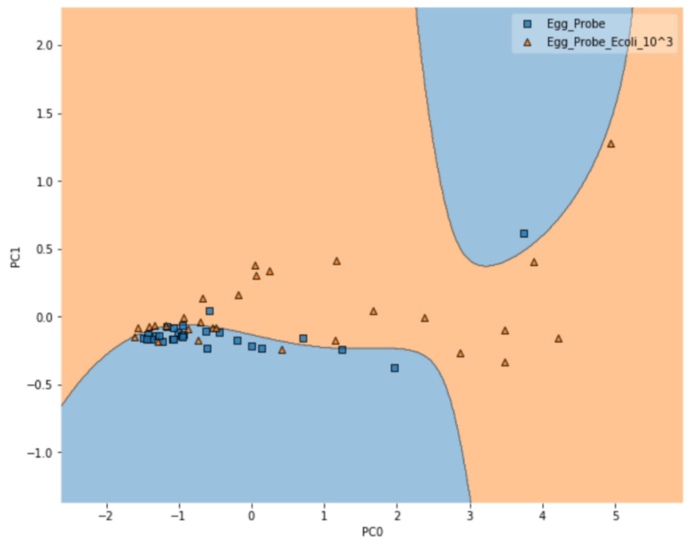


(B)

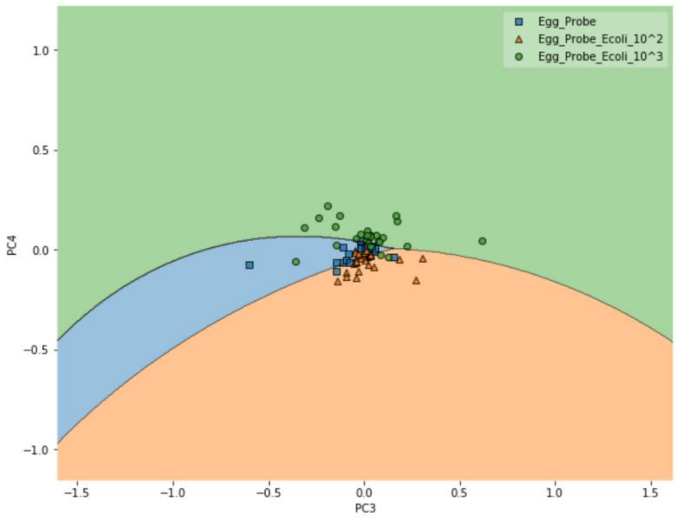


(C)

**Figure S3.** Classification of spiked vs. un-spiked egg powder using PCA-SVM discriminant modeling. (A). Contamination level of $10^2$ CFU/g; (B). Contamination level of $10^3$ CFU/g; (C). Differentiation among control, $10^2$ CFU/g and $10^3$ CFU/g samples.

These PC0/PC1 plots showed a projection of 5-D image onto a 2-D plane. Clustering of groups shown hence was not a true reflection of the separation in the 5-D space (PC0-PC4) between the groups. Nonetheless, the differentiation results for the egg powder samples were not as good as that of the black pepper powder samples. Further study is needed to understand the reason of this.

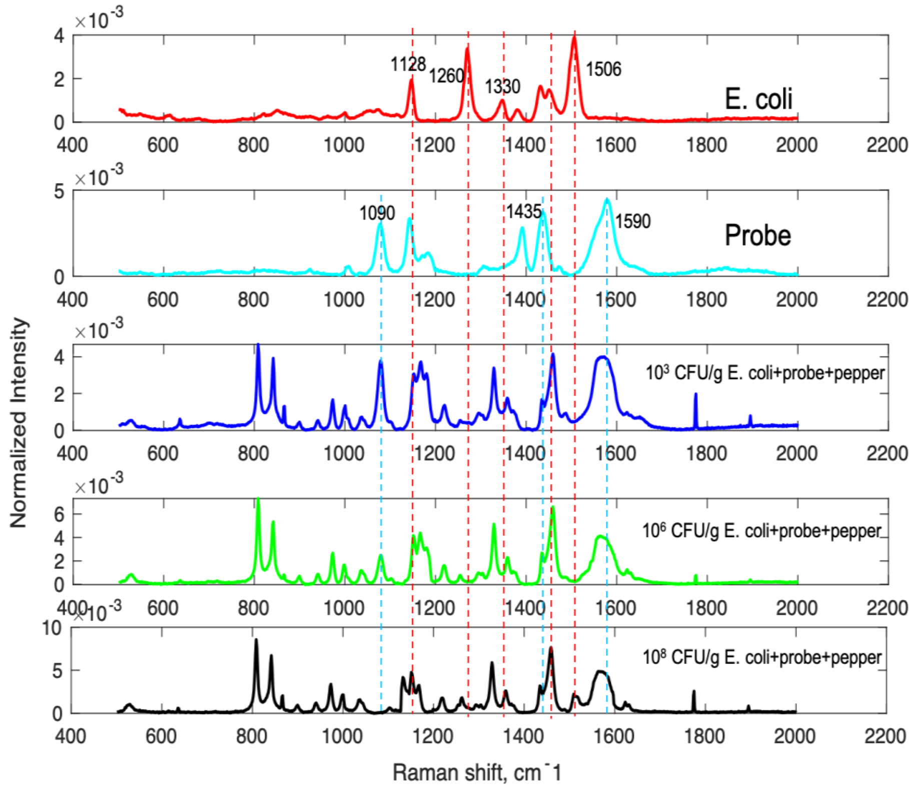


**Figure S4.** Detection of E. coli in black pepper at different contamination levels with SERS probe.

Fig. S4 shows at the cotamination levels at $10^{3\text{-}6}$ CFU/g, the spectra collected from the spiked black pepper powder samples were showing similar signatures, with peak at 1128 $cm^{-1}$ appearing to be cell originated, and peaks at 1090, 1435 and 1590 $cm^{-1}$ appearing to be probe originated. However, simple visual inspection could not be conclusive. As discussed in the main text, discriminant analysis was needed to provide a reliable detection of the bacteria, mainly due to the strong inteference of the LMF itself. As the bacteria level was further increased to $10^8$ CFU/g, peak shift in the sample spectrum was observed, most obviously the triple peaks around 1128 $cm^{-1}$ were blue-shifted, also the peak at ~1330 $cm^{-1}$ was blue-shifted to indicating influence of the cell wall carbohydrat. Most notably, a strong SERS peak of the E. coli cells (~1506 $cm^{-1}$), which has been reported for a different bacterium, *S. aureus*, to be cell wall component of carotene [7], showed up in the spectrum of the spiked sample. In addition, the probe peak at 1090 $cm^{-1}$ all but disappeared, and the 1590 $cm^{-1}$ became weakened, suggesting at high concentration of E. coli, probes may not be interacting with all cells (same amount of probes were used), and some of the probe may even be blocked by cells to result in a lowered probe signal. Nonetheless, at high concentration, visual inspection of the spectrum seemed to be sufficient to provide a definitive detection result. However, such high level of contamination rarely happens in real world. Detection at $10^2$-$10^3$ CFU/g is more paractically important.

## Reference

1. Rycenga, M.; Kim, M. H.; Camargo, P. H. C.; Cobley, C.; Li, Z.; Xia, Y. Surface-Enhanced Raman Scattering: Comparison of Three Different Molecules on Single-Crystal Nanocubes and Nanospheres of Silve. *J. Phs. Chem. A.* **2009**, *113(16)*, 3932–3939.
2. Frey, S.; Stadler, V.; Heister, K.; Eck, W.; Zharnikov, M.; Grunze, M.; Zeysing, B.; Tertort, A. Structure of Thioaromatic Self-Assembled Monolayers on Gold and Silver. *Langmuir* **2001**, *17(8)*, 2408–2415.

3. Zheng, J.; Zhou, Y.; Li, X.; Ji, Y.; Lu, T.; Gu, R. Surface-Enhanced Raman Scattering of 4-Aminothiophenol in Assemblies of Nanosized Particles and the Macroscopic Surface of Silver. *Langmuir* **2003**, *19(3)*, 632–636
4. Jiao, L.; Niu, L.; Shen, J.; You, T.; Dong, S.; Ivaska, A. Simple azo derivatization on 4-aminothiophenol/Au monolayer. *Electrochem. Comm.* **2005**, *7(2)*, 219–222.
5. Xiao, N; Yu, C. Rapid-response and highly sensitive non-crosslinking colorimetric nitrite sensor using 4-aminothiophenol modified gold nanorods. *Anal. Chem.* **2010**, *82*, 3659–3663
6. Bizzarri, A. R.; Cannistraro, S. SERS detection of thrombin by protein recognition using functionalized gold nanoparticles. *Nanomed.* **2007**, *3(4)*, 306–310.
7. Mosier-Boss P. A. Review on SERS of Bacteria. *Biosensors*. **2017**, *7(4)*, 51.

© 2020 by the authors. Licensee MDPI, Basel, Switzerland. This article is an open access article distributed under the terms and conditions of the Creative Commons Attribution (CC BY) license (http://creativecommons.org/licenses/by/4.0/).
